# Structural and functional investigation of the human snRNP assembly factor AAR2 in complex with the RNase H-like domain of PRPF8

**DOI:** 10.1107/S2059798322009755

**Published:** 2022-10-27

**Authors:** Marco Preussner, Karine F. Santos, Jonathan Alles, Christina Heroven, Florian Heyd, Markus C. Wahl, Gert Weber

**Affiliations:** a Laboratory of RNA Biochemistry, Freie Universität Berlin, Takustrasse 6, 14195 Berlin, Germany; b Laboratory of Structural Biochemistry, Freie Universität Berlin, Takustrasse 6, 14195 Berlin, Germany; c Macromolecular Crystallography, Helmholtz-Zentrum Berlin für Materialien und Energie, Albert-Einstein-Strasse 15, 12489 Berlin, Germany; University of Western Australia, Crawley, Australia

**Keywords:** spliceosomal assembly, U5 snRNP, AAR2, PRPF8

## Abstract

Small nuclear ribonucleoprotein complexes (snRNPs) represent the main subunits of the spliceosome. While the assembly of the snRNP core particles has been well characterized, comparably little is known of the incorporation of snRNP-specific proteins and the mechanisms of snRNP recycling. U5 snRNP assembly in yeast requires binding of the the Aar2 protein to Prp8p as a placeholder to preclude premature assembly of the SNRNP200 helicase, but the role of the human AAR2 homolog has not yet been investigated in detail. Here, a crystal structure of human AAR2 in complex with the RNase H-like domain of the U5-specific PRPF8 (PRP8F RH) is reported, revealing a significantly different interaction between the two proteins compared with that in yeast. Based on the structure of the AAR2–PRPF8 RH complex, the importance of the interacting regions and residues was probed and AAR2 variants were designed that failed to stably bind PRPF8 *in vitro*. Protein-interaction studies of AAR2 with U5 proteins using size-exclusion chromatography reveal similarities and marked differences in the interaction patterns compared with yeast Aar2p and imply phosphorylation-dependent regulation of AAR2 reminiscent of that in yeast. It is found that *in vitro* AAR2 seems to lock PRPF8 RH in a conformation that is only compatible with the first transesterification step of the splicing reaction and blocks a conformational switch to the step 2-like, Mg^2+^-coordinated conformation that is likely during U5 snRNP biogenesis. These findings extend the picture of AAR2 PRP8 interaction from yeast to humans and indicate a function for AAR2 in the spliceosomal assembly process beyond its role as an SNRNP200 placeholder in yeast.

## Abstract 2

The crystal structure of human AAR2 bound to the central spliceosomal factor PRPF8 and *in vitro* functional data yield insights into the structural basis of snRNP assembly in humans.

## 1. Introduction

Precursor messenger RNA (pre-mRNA) splicing is catalyzed by a highly dynamic, multi-megadalton ribonucleoprotein (RNP) machinery, the spliceosome (Will & Lührmann, 2011 ▸; Wahl *et al.*, 2009 ▸). The small nuclear RNPs (snRNPs) U1, U2, U4, U5 and U6 are the main subunits of the major, U2-type spliceosome. Each of these snRNPs contains a particle-specific snRNA, seven common Sm proteins [or like Sm (LSm) proteins in the case of U6] and a set of particle-specific proteins (Will & Lührmann, 2001 ▸). The U5 snRNP is the only snRNP subunit that is also employed by the minor spliceosome (Will & Lührmann, 2005 ▸). Apart from the snRNPs, a multitude of proteins and protein complexes that are not stably associated with an snRNP also join the spliceosome to facilitate and regulate pre-mRNA splicing (Agafonov *et al.*, 2011 ▸). For each round of splicing, the spliceosome assembles anew on a pre-mRNA by the stepwise association of snRNPs and non-snRNP proteins. Its catalytic center is not preformed but only emerges during assembly by repeated, extensive remodeling of the specific RNA–protein interaction network of each assembly stage to eventually elicit intron excision and exon ligation via two transesterification reactions, referred to as step 1 and step 2 (Wahl *et al.*, 2009 ▸; Will & Lührmann, 2011 ▸).

SnRNPs themselves are assembled via complex pathways, which in the cases of U1, U2, U4 and U5 include cytoplasmic and nuclear phases (Will & Lührmann, 2001 ▸; Matera & Wang, 2014 ▸; Gruss *et al.*, 2017 ▸). The corresponding snRNAs are synthesized by RNA polymerase II (Pol II), modified with an m^7^G cap, processed by the integrator complex and exported to the cytoplasm. Here, the Sm proteins are assembled stepwise to form a ring-like structure around a U-rich Sm site in the snRNAs via the protein arginine methyltransferase 5 complex and the survival motor neuron (SMN) complex. Trimethylguanosine synthase 1 then catalyzes hypermethylation of the m^7^G cap to generate an m^2,2,7^G cap. The hypermethylated cap and the assembled Sm core domain act as a composite nuclear localization signal that facilitates re-entry of the Sm core RNPs into the nucleus.

The integration of particle-specific proteins into snRNPs also requires specific assembly factors and chaperones. For example, in human cells, the adaptor protein, nuclear FMR1-interacting protein 1, and the heat-shock protein 90 (HSP90)/Rvb1–Rvb2–Tah1–Pih1 (RT2P) chaperone machinery in collaboration with a nuclear-localized SMN complex facilitate integration of the U4/U6-specific proteins NHP2-like protein 1 and pre-mRNA processing factor (PRPF) 31 into the U4/U6 di-snRNP (Bizarro *et al.*, 2015 ▸). The HSP90/RT2P chaperone machinery also supports the assembly of a U5 snRNP module composed of the PRPF8 protein (Prp8p in yeast), the 116 kDa U5 small nuclear ribonucleoprotein component (EFTUD2; Snu114p in yeast), the U5 small nuclear ribonucleoprotein 200 kDa helicase (SNRNP200; Brr2p in yeast) and the SNRNP40 protein in the cytoplasm, thereby promoting formation of the mature U5 snRNP (Malinová *et al.*, 2017 ▸). Several additional proteins have been implicated in U5 snRNP or U4/U6-U5 tri-snRNP assembly in metazoans (Malinová *et al.*, 2017 ▸; Klimešová *et al.*, 2021 ▸; Erkelenz *et al.*, 2021 ▸; Bergfort *et al.*, 2022 ▸).

Some snRNPs are profoundly remodeled during pre-mRNA splicing, necessitating specific recycling mechanisms to reassemble the particles for further rounds of splicing. For example, during spliceosome activation the U4 and U6 snRNAs, which are extensively base-paired in the U4/U6 di-snRNP, are unwound by the SNRNP200 helicase, and U4 snRNA and all U4/U6-associated proteins are displaced (Laggerbauer *et al.*, 1998 ▸; Raghunathan & Guthrie, 1998 ▸; Agafonov *et al.*, 2011 ▸). Furthermore, in human cells the U5 snRNP enters the spliceosome as a 20S particle but is released as a 35S particle after splicing due to incorporation of the PRPF19 complex and additional factors (Makarov *et al.*, 2002 ▸). Late *de novo* snRNP biogenesis steps and recycling of snRNPs after pre-mRNA splicing take place in nuclear Cajal bodies (Staněk & Neugebauer, 2004 ▸, 2006 ▸; Sleeman *et al.*, 2001 ▸; Sleeman & Lamond, 1999 ▸).

The U5-specific PRPF8 protein is one of the most conserved nuclear proteins and coordinates proteins, snRNAs and the pre-mRNA at the catalytic center of the spliceosome (Grainger & Beggs, 2005 ▸). PRPF8 harbors two regulatory pseudo-enzyme domains at its very C-terminus, comprising an RNase H-like (RH) and a Jab1/MPN-like (JM) fold (Pena *et al.*, 2007 ▸, 2008 ▸; Zhang *et al.*, 2007 ▸; Yang *et al.*, 2008 ▸; Ritchie *et al.*, 2008 ▸). In yeast, the A1 cistron-splicing factor (Aar2p) has been characterized as a U5 snRNP assembly and recycling factor that mediates the formation of pre-U5 snRNPs lacking the Brr2p helicase in the cytoplasm (Gottschalk *et al.*, 2001 ▸; Boon *et al.*, 2007 ▸). Aar2p concomitantly binds the RH and JM domains of Prp8p (Weber *et al.*, 2011 ▸, 2013 ▸; Galej *et al.*, 2013 ▸). By sequestering the Prp8p JM domain, which is a major binding site of the Brr2p RNA helicase, Aar2p initially prevents the integration of Brr2p into U5 snRNP (Weber *et al.*, 2013 ▸; Galej *et al.*, 2013 ▸). Aar2p accompanies the Brr2p-deficient pre-U5 snRNP particle into the nucleus, where phosphorylation of its Ser253 residue triggers refolding and release of Aar2p, allowing Brr2p entry to complete U5 snRNP biogenesis (Boon *et al.*, 2007 ▸; Weber *et al.*, 2013 ▸).

In a previous study, we demonstrated that the human Aar2p homolog AAR2 is produced from the *c20orf4* gene in HeLa cells and that it stably binds the PRPF8 RH domain *in vitro* (Santos *et al.*, 2015 ▸). However, human AAR2 and yeast Aar2p exhibit only 24% sequence identity, questioning the extent to which their structures and molecular mechanisms are conserved. While proteomics studies and pull-down experiments have suggested that human AAR2 also participates in U5 snRNP assembly (Malinová *et al.*, 2017 ▸; Klimešová *et al.*, 2021 ▸), human AAR2 has been identified in a complex with PRPF8, EFTUD2, SNRNP200 and SNRNP40 (Malinová *et al.*, 2017 ▸), indeed indicating potential differences in the Aar2p/AAR2-mediated U5 snRNP assembly steps in yeast and humans.

Here, we report a co-crystal structure of human AAR2 in complex with the PRPF8 RH domain (PRPF8^RH^) and present further interaction studies of AAR2 with PRPF8 fragments and the SNRNP200 helicase *in vitro*. In contrast to the situation in yeast, we find that a human AAR2–PRPF8^RH^ complex does not bind the PRPF8 JM domain and thus permits the formation of a trimeric AAR2–PRPF8–SNRNP200 complex. As in yeast, the human AAR2–PRPF8^RH^ interaction is abrogated *in vitro* by a phosphomimetic S284E (S253E in yeast) mutation, indicating highly conserved regulation of AAR2 by phosphorylation. Furthermore, AAR2 seems to lock PRPF8^RH^ in its first-step conformation and block the conformational switch to a step 2-like, Mg^2+^-coordinated conformation during U5 snRNP biogenesis. Our results shed the first light on the human AAR2–PRPF8^RH^ interface and imply a different role of AAR2 in spliceosomal assembly than in yeast.

## 2. Materials and methods

### 2.1. Cloning, expression, protein purification and reconstitution of protein complexes

We employed a modified pFL vector encoding a truncated version of human SNRNP200 lacking the first 394 residues (residues 395–2136) and containing an N-terminal, TEV-cleavable His_10_ tag (Santos *et al.*, 2012 ▸), a pET-M11 plasmid encoding human PRPF8^RH^ (residues 1747–2016) and containing an N-terminal, TEV-cleavable His_6_ tag (Pena *et al.*, 2008 ▸; Weber *et al.*, 2011 ▸), a pET-M11 plasmid encoding human PRPF8^JM^ (residues 2064–2335) and containing an N-terminal TEV-cleavable GST tag (Mozaffari-Jovin *et al.*, 2013 ▸), and pFL vectors encoding human AAR2 or AAR2^Δloop^ (AAR2 with residues 170–200 replaced by three serines) with both AAR2 constructs containing an N-terminal, TEV-cleavable His_10_ tag (Santos *et al.*, 2015 ▸), which have been described previously. The inserts were derived from codon-optimized synthetic genes. A DNA fragment encoding the C-terminal fragment of PRPF8 (encompassing the RH and JM domains; PRPF8^RH-JM^; residues 1760–2335) was PCR-amplified from a codon-optimized *prpf8* synthetic gene and cloned into the pIDK donor vector by restriction-enzyme cloning using XhoI and NdeI (NEB). *Snrnp200^395–2136^*-pFL and *prpf8^1760–2335^*-pIDK were fused by Cre-Lox recombination and used for bacmid preparation. A codon-optimized DNA fragment encoding human AAR2 residues 1–364, lacking the C-terminal 20 residues of AAR2, was cloned into a modified pFL vector by restriction-enzyme cloning using EcoRI and HindIII (NEB) to guide the production of AAR2^1–364^ containing a C-terminal His_6_ tag. A codon-optimized DNA fragment encoding human AAR2 was cloned into pcDNA3.1(+) using BamHI and XhoI restriction enzymes (NEB) to guide the production of AAR2 containing an N-terminal FLAG tag. Mutations were introduced with the QuikChange site-directed mutagenesis kit (Stratagene) according to the manufacturer’s instructions. All constructs were verified by dye-terminator sequencing (Seqlab).

Purified human SNRNP200^395–2136^ (8 mg ml^−1^; Santos *et al.*, 2012 ▸), PRPF8^RH^ (30 mg ml^−1^; Pena *et al.*, 2008 ▸), PRPF8^JM^ (10 mg ml^−1^; Mozaffari-Jovin *et al.*, 2013 ▸), AAR2 (12 mg ml^−1^; Santos *et al.*, 2015 ▸), AAR2^Δloop^ (12 mg ml^−1^; Santos *et al.*, 2015 ▸), an SNRNP200^395–2136^–PRPF8^JM^ complex (Mozaffari-Jovin *et al.*, 2013 ▸; 8 mg ml^−1^) and an AAR2–PRPF8^RH^ complex (20 mg ml^−1^; Santos *et al.*, 2015 ▸) were obtained as described previously. AAR2^1–364^ (11 mg ml^−1^) was produced and purified as described for AAR2 but with omission of the TEV protease cleavage step, leaving the His_6_ tag intact. SNRNP200^395–2136^ and PRPF8^RH-JM^ were co-produced based on a recombinant baculovirus derived from the recombined bacmid in Sf9 cells. For purification of the SNRNP200^395–2136^–PRPF8^RH-JM^ complex, cells were resuspended in resuspension buffer [50 m*M* Tris–HCl pH 8.0, 300 m*M* NaCl, 5%(*v*/*v*) glycerol, 1 m*M* DTT, 0.05%(*v*/*v*) NP40] supplemented with EDTA-free protease inhibitors (Roche) and DNase I (NEB) and lysed via sonification. After centrifugation, the lysate of about 50 column volumes was filtered and passed through Ni–NTA beads (Qiagen). The beads were washed twice with ten column volumes of resuspension buffer containing 15 m*M* imidazole. The captured complex was eluted with two column volumes of resuspension buffer containing 500 m*M* imidazole, TEV protease (0.5 mg per millitre of protein solution) was added and the mixture was dialyzed against 20 m*M* HEPES–NaOH pH 7.5, 200 m*M* NaCl, 1 m*M* DTT overnight at 4°C. Five column volumes of the buffer-exchanged sample were again passed through Ni–NTA beads and the flowthrough was collected. The complex was concentrated to a final concentration of 7 mg ml^−1^ and further purified by size-exclusion chromatography (SEC) on a Superdex S200 10/300 column (GE Healthcare) in 20 m*M* HEPES–NaOH pH 7.5, 200 m*M* NaCl, 1 m*M* DTT.

### 2.2. Analytical size-exclusion chromatography

Individual proteins and protein mixtures were analyzed by analytical SEC on a Superdex 200 Increase PC3.2/30 column (GE Healthcare) in 20 m*M* Tris–HCl pH 7.5, 250 m*M* NaCl, 0.5 m*M* DTT at a flow rate of 50–70 µl min^−1^. For analysis of complex formation, proteins (at the concentrations stated in Section 2.1) were mixed in equimolar ratios in 60 µl size-exclusion buffer and incubated for 30 min on ice. Elution fractions were supplemented with SDS–PAGE loading buffer and analyzed by SDS–PAGE.

### 2.3. Crystallographic analyses

Crystallization of the human AAR2^Δloop^–PRPF8^RH^ complex has been described previously (Santos *et al.*, 2015 ▸). Briefly, 1 µl purified human AAR2^Δloop^–PRPF8^RH^ complex concentrated to 14 mg ml^−1^ in 20 m*M* Tris–HCl pH 7.5, 150 m*M* NaCl, 0.5 m*M* DTT was crystallized employing an equal volume of reservoir solution consisting of 0.1 *M* HEPES pH 7.0, 10%(*w*/*v*) PEG 6000, 5%(*v*/*v*) 2-methyl-2,4-pentanediol. Crystals were transferred to reservoir solution supplemented with 10%(*v*/*v*) 2-methyl-2,4-pentanediol and flash-cooled in liquid nitrogen. Diffraction data were collected on beamlines BL14.1, BL14.2 and BL14.3 of the BESSY II storage ring, Berlin, Germany and on beamline P14 of the PETRA III storage ring, Hamburg, Germany at 100 K and were processed with *XDS* (Kabsch, 2010 ▸). The structure was solved by molecular replacement with *Phaser* (McCoy *et al.*, 2007 ▸) using chain *A* of the structural coordinates of human PRPF8RH (PDB entry 3e9l; Pena *et al.*, 2008 ▸), omitting the water molecules. An initial model of the AAR2^Δloop^ subunit was obtained by automated model building with *phenix.autobuild* (Adams *et al.*, 2010 ▸). The model was completed through alternating rounds of automated refinement using *phenix.refine* (Afonine *et al.*, 2012 ▸) and manual model building using *Coot* (Emsley *et al.*, 2010 ▸). The quality of the final model was assessed with *MolProbity* (Chen *et al.*, 2010 ▸). Of note, a relatively large number of difference density peaks were observed in the *F*_o_ − *F*_c_ map. Structural figures were prepared with *PyMOL* (Schrödinger).

## 3. Results

### 3.1. Crystal structure of a human AAR2^Δloop^–PRPF8^RH^ complex

Structures of yeast Aar2p in complex with the Prp8p RH and JM domains or with full-length Prp8p have been reported (Weber *et al.*, 2011 ▸, 2013 ▸; Galej *et al.*, 2013 ▸). In contrast, the structure of human AAR2, which exhibits only 24% sequence identity to the yeast ortholog (Supplementary Fig. S1), and the structural basis for its interaction with PRPF8 remain unknown. We previously reported the crystallization of a human AAR2 variant in which an internal loop (residues 170–200) was replaced by three serine residues (AAR2^Δloop^) in complex with PRPF8^RH^ (Santos *et al.*, 2015 ▸). In yeast Aar2p, the corresponding internal loop was shown to hinder crystallization, to be protease-cleavable and to be irrelevant for the interaction with Prp8p C-terminal domains (Weber *et al.*, 2011 ▸, 2013 ▸; Santos *et al.*, 2015 ▸). Here, we report the crystal structure of the human AAR2^Δloop^–PRPF8^RH^ complex at 2.35 Å resolution. The structure was solved by molecular replacement with *Phaser* using the structural coordinates of PRPF8^RH^ (PDB entry 3e9l; Pena *et al.*, 2008 ▸) as a molecular-replacement search model (Table 1 ▸).

Apart from ten N-terminal residues, the region spanning residues 159–201 including the three serines connecting residues 170–200 of the internal loop, a loop connecting helices α9 and α10 (residues 313–321) and six C-terminal residues of AAR2^Δloop^ (Supplementary Fig. S1), all residues of AAR2^Δloop^ and PRPF8^RH^ could be reliably modeled into well defined electron density (Supplementary Fig. S2). Residues 65–71 of AAR2 and residues 2001–2008 of PRPF8^RH^ were modeled with low confidence due to weaker electron density.

**Figure 1:**
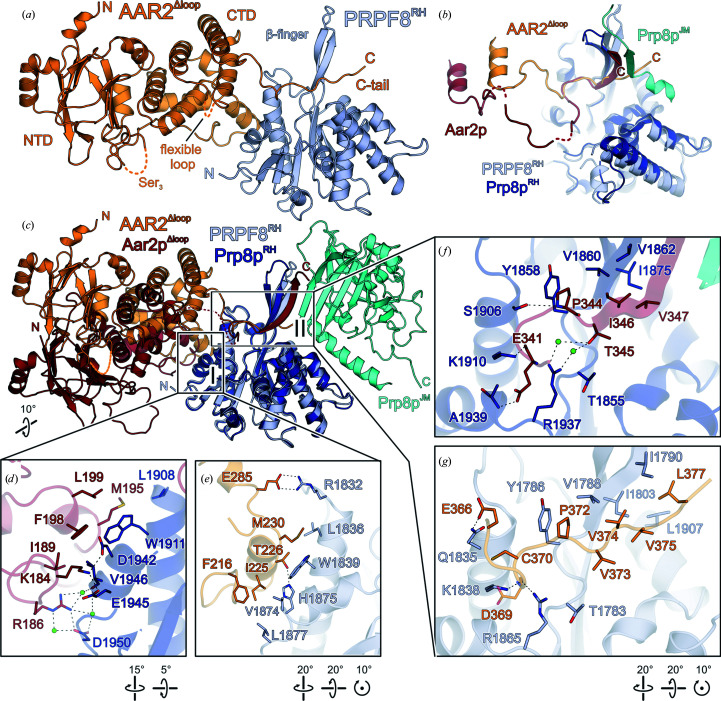
(*a*) Crystal structure of the human AAR2^Δloop^–PRPF8^RH^ complex. Colour scheme for this and the following figures: AAR2^Δloop^, orange; PRPF8^RH^, light blue; dashed orange lines indicate a flexible loop (labeled Ser_3_) in AAR2 connecting its two domains, which is replaced by three serine residues in AAR2^Δloop^ (Santos *et al.*, 2015 ▸), and another smaller flexible loop between residues 313 and 321 (labeled flexible loop). The N- and C-termini as well as the β-finger module of PRPF8^RH^ are labeled. (*b*) Superposition of the RH domains of human PRPF8 and yeast Prp8p in complex with human AAR2^Δloop^ and yeast Aar2p/PRPF8^JM^ (PDB entry 4i43; Galej *et al.*, 2013 ▸), respectively, to illustrate the human AAR2 in a larger PRPF8 context. Colour scheme for this and the following figures: Aar2p, maroon; Prp8p^RH^, dark blue; Prp8p^JM^, cyan. (*c*) Comparison of the human AAR2^Δloop^–PRPF8^RH^ complex and the yeast Aar2p^Δloop^–Prp8p^RH^–Prp8p^JM^ complex (PDB entry 4ilg; Weber *et al.*, 2013 ▸) by superposition of the RH domains. Dashed maroon line, flexible linker preceding the C-terminal tail of Aar2p (Weber *et al.*, 2013 ▸). (*d*, *e*) Close-up views of interface regions I of the yeast Aar2p^Δloop^–Prp8p^RH^–Prp8p^JM^ complex (*d*) and the human AAR2^Δloop^–PRPF8^RH^ complex (*e*). (*f*, *g*) Close-up views of interface regions II of the yeast Aar2p^Δloop^–Prp8p^RH^–Prp8p^JM^ complex (*f*) and the human AAR2^Δloop^–PRPF8^RH^ complex (*g*). In (*d*–*f*) and the following figures interacting residues are shown as sticks colored by atom type, with carbon colored as the respective protein, nitrogen in blue, oxygen in red and sulfur in yellow; green spheres are water molecules, dashed black lines represent hydrogen bonds or salt bridges and rotation symbols represent orientations relative to (*a*).

Despite the low sequence identity, the overall structure of human AAR2^Δloop^ in the AAR2^Δloop^–PRPF8^RH^ complex is very similar to that of yeast Aar2p^Δloop^ in complex with the Prp8p RH and JM domains (Weber *et al.*, 2013 ▸; Galej *et al.*, 2013 ▸; root-mean-square deviation of 2.13 Å for 236 pairs from 330 AAR2 and 342 Aar2p C^α^ atoms; Supplementary Fig. S3). As previously observed for Aar2p (Weber *et al.*, 2011 ▸, 2013 ▸; Galej *et al.*, 2013 ▸), human AAR2^Δloop^ exhibits an N-terminal domain (NTD; residues 10–158) mainly composed of β-strands, an α-helical C-terminal domain (CTD; residues 202–364) and a C-terminal, irregularly structured tail (residues 365–384) (Fig. 1 ▸ *a*).

In the full-length yeast Aar2p–Prp8p structure (PDB entry 4i43; Galej *et al.*, 2013 ▸), the C-terminal peptide of Aar2p is fully structured and contacts several other Prp8p domains. Superposition with the present human AAR2–RH complex structure suggests that due to the shorter AAR2 C-terminal peptide in humans, contacts with other PRPF8 domains may be limited. Hence, the differences in the functionally important C-terminal peptide of yeast and human Aar2p/AAR2 may hint at a somewhat different mode of action of AAR2 in U5 snRNP or U4/U6-U5 tri-snRNP assembly in humans.

Despite the overall structural similarity of both individual components, the protein interfaces between yeast and human Prp8p^RH^/PRPF8^RH^ and Aar2p/AAR2 are markedly different. As in yeast, the NTD lacks direct interactions with PRPF8^RH^, while the CTD and the C-terminal tail of AAR2 establish two interfaces with PRPF8^RH^ (interfaces I and II, respectively; Figs. 1 ▸ *a*–1 ▸ *c*). In interface I, an edge of the AAR2^Δloop^ CTD laterally contacts PRPF8^RH^ (Figs. 1 ▸ *d* and 1 ▸ *e*). Interface II is built by the C-terminal residues 366–377 of AAR2 extending across the PRPF8 RH domain below the protruding β-finger module (Figs. 1 ▸ *f* and 1 ▸ *g*). Both interfaces bury a comparable surface area in the yeast and human systems (interface I, 399 and 412 Å^2^, respectively; interface II, 733 and 511 Å^2^, respectively).

Interface I is dominated by hydrophobic contacts, with only four of 12 PRPF8^RH^-interacting residues conserved between yeast Aar2p and human AAR2, underlining the different organization of the interactions. The conserved core of interface I includes interactions between Ile225 of AAR2 (Ile189 in Aar2p) and Val1874 of PRPF8^RH^ (Val1946 in Aar2p) as well as between Met230 of AAR2 (Met195 in Aar2p) and Trp1839 of PRPF8^RH^ (Trp1911 in Aar2p) (Figs. 1 ▸ *d* and 1 ▸ *e*; Supplementary Fig. S1). Compared with yeast Aar2p, the AAR2 CTD harbors two extended helices (α11 and α12; Supplementary Fig. S3). In addition, Ile225 and Met230 are shifted by four residues (about one helical turn) along the α5 helix compared with the equivalent residues in yeast Aar2p (Figs. 1 ▸ *d* and 1 ▸ *e*), giving rise to a markedly different angle with which human AAR2^Δloop^ contacts the PRPF8 RH domain compared with yeast Aar2p^Δloop^ in the Aar2p^Δloop^–Prp8p^RH^–Prp8p^JM^ complex (Fig. 1 ▸ *c*). Also, the AAR2^Δloop^ residues participating in interface II are only partially conserved between yeast and humans (two of eight residues; Supplementary Fig. S1).

### 3.2. Similarities and differences in AAR2–PRPF8–SNRNP200 and Aar2p–Prp8p–Brr2p interactions in humans and yeast

**Figure 2:**
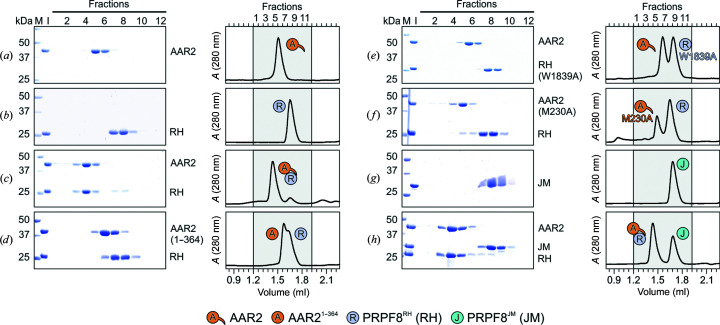
Probing interacting regions and residues in AAR2^Δloop^–PRPF8^RH^. (*a*–*h*) SDS–PAGE analyses (left) and UV elution profiles (right) of analytical size-exclusion chromatography runs monitoring the interactions among AAR2 variants, PRPF8^RH^ variants and PRPF8^JM^. (*a*)–(*c*) were adapted from Santos *et al.* (2015 ▸) and are shown for comparison. Lane M, molecular-mass standard (kDa); lane I, input samples. Protein bands are identified on the right. Elution fractions are indicated at the top of the gels and profiles; elution volumes are indicated at the bottom of the profiles. Icons are explained at the bottom. Variants are indicated below the respective icons. Peaks labeled by transparent icons represent an excess of the respective protein.

The low degree of conservation of AAR2 and observed marked differences in the interface with PRPF8^RH^ have apparent consequences for AAR2 function and likely for interactions within the spliceosome. To test the importance of the specific contacts between AAR2^Δloop^ and PRPF8^RH^ that are observed in our co-crystal structure, we conducted analytical SEC runs with wild-type (WT) proteins and variants. To this end, we investigated the binding of WT AAR2 to WT PRPF8^RH^ in previous work, which is only shown here for comparison (Figs. 2 ▸ *a*–2 ▸ *c*; Santos *et al.*, 2015 ▸). In yeast, the C-terminal tail of Aar2p is dispensable for Prp8^RH^ binding (Weber *et al.*, 2011 ▸). Conversely, in the human system, AAR2^1–364^, which lacks the C-terminal tail, no longer binds stably to PRPF8^RH^ (Figs. 2 ▸ *a*–2 ▸ *d*). Likewise, converting Trp1839 of PRPF8^RH^ or Met230 of AAR2, which are part of the conserved core of interface I, individually to alanine residues abrogated complex formation (Fig. 2 ▸ *e* and 2 ▸ *f*). Again, the situation differs in yeast, where only Trp1911 of Prp8p^RH^ (equivalent to Trp1839 in human PRPF8^RH^), but not Met195 of Aar2p (equivalent to Met230 in human AAR2), is essential for the interaction (Weber *et al.*, 2013 ▸).

The low sequence conservation and the resulting structural differences in the AAR2–PRP8^RH^ interface might also have consequences for the wider protein interaction network around AAR2. Concomitant binding of the Prp8p RH and JM domains by Aar2p in yeast sequesters the JM domain, preventing binding of the Brr2p RNA helicase to Aar2p–pre-U5 snRNP (Weber *et al.*, 2013 ▸; Galej *et al.*, 2013 ▸). In yeast Aar2p–Prp8p complexes (Weber *et al.*, 2011 ▸, 2013 ▸; Galej *et al.*, 2013 ▸), the C-terminal tail of Aar2p runs along the protruding Prp8p^RH^ β-finger module, stringing the β-finger and the central Prp8p^JM^ β-sheet into an extended, intermolecular β-structure (Figs. 1 ▸ *b* and 1 ▸ *c*).

While the beginning of the C-terminal tail in human AAR2^Δloop^ maintains similar interactions with PRPF8^RH^ as in yeast, for example employing Val373–Val375 to form a short β-sheet of three hydrogen bonds to PRPF8^RH^, distal parts of the C-terminal tail (beyond Val374) deviate from the direction of the Aar2p C-terminal tail (Figs. 1 ▸ *b* and 1 ▸ *c*). In yeast, the formation of the penultimate β-strand of Aar2p and the concomitant sequestration of JM from Brr2p is mediated exclusively by a series of hydrophobic residues at the very C-terminus of Aar2p, which are complementary to hydrophobic residues of the neighboring β-strands of RH and JM (Weber *et al.*, 2013 ▸; Galej *et al.*, 2013 ▸). A structure-based alignment revealed that the respective very C-terminal residues of AAR2, Pro378, Glu379, Gly380 and Glu382, are unlikely to support β-sheet formation with the corresponding highly conserved residues of the PRPF8^RH^ β-finger and PRPF8^JM^ due to their steric or polar properties (Supplementary Fig. S1; compare Figs. 1 ▸ *f* and 1 ▸ *g*). However, we cannot exclude that in the context of the full-length proteins the very C-terminus of hAAR2 may engage in a yeast-like interaction with the PRPF8 JM domain.

**Figure 3:**
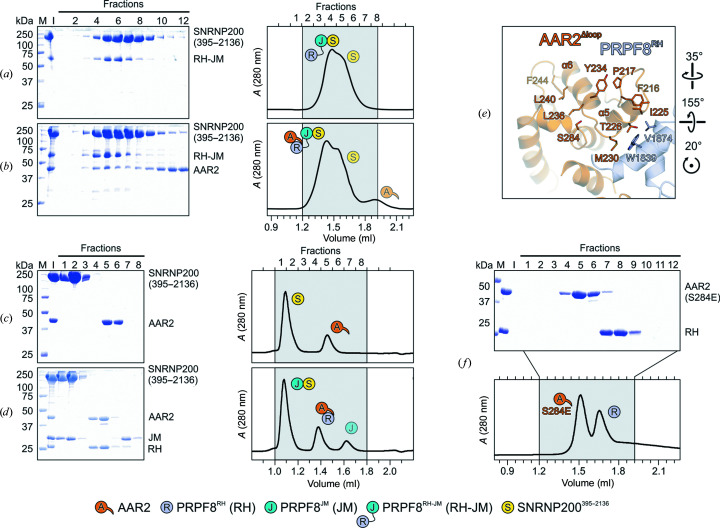
Probing AAR2^Δloop^–PRPF8–SNRNP200 interactions and AAR2 phosphorylation. (*a*–*d*) SDS–PAGE analyses (left) and UV elution profiles (right) of analytical size-exclusion chromatography runs monitoring the interactions among AAR2, PRPF8^RH-JM^ and SNRNP200^395–2136^ (*a*, *b*) and among AAR2, PRPF8^RH^, PRPF8^JM^ and SNRNP200^395–2136^ (*c*, *d*). (*e*) Close-up view of the region in AAR2^Δloop^–PRPF8^RH^ surrounding Ser284 of AAR2^Δloop^. The corresponding region in yeast Aar2p is profoundly restructured upon replacement of the equivalent Ser253 by a phosphomimetic glutamate residue (Weber *et al.*, 2013 ▸). (*f*) SDS–PAGE analysis (top) and UV elution profile (bottom) of an analytical size-exclusion chromatography run monitoring the interaction between AAR2^S284E^ and PRPF8^RH^. In panels showing SDS–PAGE gels and elution profiles lane M contains molecular-mass standard (kDa) and lane I contains input samples. Protein bands are identified on the right. Elution fractions are indicated at the top of the gels and profiles; elution volumes are indicated at the bottom of the profiles. Icons are explained at the bottom. Variants are indicated below the respective icons. Peaks labeled by transparent icons represent an excess of the respective protein.

The C-terminal tail of human AAR2^Δloop^ in the observed conformation would not be able to concomitantly bind the PRPF8^JM^ domain as observed in yeast. Indeed, also confirming a prior study (Malinová *et al.*, 2017 ▸), AAR2–PRPF8^RH^ did not stably bind PRPF8^JM^ in analytical SEC (Figs. 2 ▸ *g* and 2 ▸ *h*) and failed to sequester PRPF8^JM^ from a pre-formed SNRNP200^395–2136^–PRPF8^JM^ complex (Fig. 3 ▸ *a* and 3 ▸ *b*).

AAR2 alone or in complex with PRPF8^RH^ did not bind stably to SNRNP200^395–2136^ or to a SNRNP200^395–2136^–PRPF8^JM^ complex (Figs. 3 ▸ *c* and 3 ▸ *d*). Instead, a stable AAR2–PRPF8^RM-JM^–SNRNP200^395–2136^ ternary complex was formed upon mixing the components (Fig. 3 ▸ *b*).

### 3.3. Conserved Aar2 phosphorylation between humans and yeast

Aar2p can be phosphorylated at five positions *in vivo* (Ser253, Thr274, Tyr328, Ser331 and Thr345) and phosphomimetic S253D or S253E variants of Aar2p interfered with Aar2p–Prp8p interaction in yeast extracts (Weber *et al.*, 2011 ▸). Structural analysis of a phosphomimetic Aar2p^S253E^ variant suggested that phosphorylation leads to a local conformational rearrangement of the Aar2p CTD and thereby to disruption of the Prp8p^RH^ binding site (Weber *et al.*, 2011 ▸). A structure-based sequence alignment revealed that Ser284 in human AAR2 corresponds to Ser253 in yeast Aar2p (Supplementary Fig. S1; Fig. 3 ▸ *e*), and AAR2 has been found to be phosphorylated at Ser284 in human liver cancer cells (Hornbeck *et al.*, 2012 ▸). Recapitulating the situation in yeast, an AAR2^S284E^ phosphomimetic variant failed to stably bind PRPF8^RH^ in analytical SEC (Fig. 3 ▸ *f*). Taken together, our interaction studies reveal differences in the relative importance of AAR2/Aar2p regions in maintaining a stable interaction with the PRPF8/Prp8p RH domain in the human and yeast systems. Furthermore, AAR2 does not sequester the PRPF8 JM domain to intermittently prevent SNRNP200 association with the U5 snRNP. AAR2 displacement from PRPF8 may involve reversible phosphorylation of AAR2 at Ser284. Thus, the U5 snRNP assembly steps apparently differ in detail in yeast and humans.

### 3.4. Human AAR2 counteracts a step 2-like conformation in the PRPF8 RH domain

Mutations in the *prpf8* gene can lead to retinitis pigmentosa (RP; Růžičková & Staněk, 2017 ▸), a disease that causes blindness in humans, and the corresponding PRPF8/Prp8p variants cause defects in U5 snRNP assembly (Malinová *et al.*, 2017 ▸) and splicing (Mayerle & Guthrie, 2016 ▸; Mozaffari-Jovin *et al.*, 2013 ▸) in humans and yeast. In baker’s yeast, two sets of *prp8* mutant alleles, corresponding to RP-related mutations in humans that disrupt either the first or the second step of splicing, cluster in the Prp8p RH domain (Grainger & Beggs, 2005 ▸).

Furthermore, the human PRPF8 RH domain can undergo a conformational switch in a protruding β-finger module, with one conformation promoting the first step and an alternative, Mg^2+^-bound conformation supporting the second step of splicing (Schellenberg *et al.*, 2013 ▸). Despite the biochemical and structural evidence reported previously, which supports this switch, a caveat of our AAR2–PRPF8^RH^ structure may be that the RH β-finger module makes crystal contacts with a neighboring symmetry-related RH β-finger module. However, recent cryogenic electron-microscopy structures of spliceosomes also confirm this conformational switch, rationalize some of the effects of PRPF8 RH domain variants and demonstrate repeated, long-range repositioning of the PRPF8/Prp8p RH domain during the splicing reaction in yeast and humans (Wan *et al.*, 2016 ▸; Bertram, Agafonov, Liu *et al.*, 2017 ▸; Yan *et al.*, 2015 ▸, 2017 ▸; Zhang *et al.*, 2017 ▸; Bertram, Agafonov, Dybkov *et al.*, 2017 ▸; Rauhut *et al.*, 2016 ▸; Galej *et al.*, 2016 ▸; Plaschka *et al.*, 2017 ▸; Fica *et al.*, 2017 ▸; Wilkinson *et al.*, 2021 ▸).

**Figure 4:**
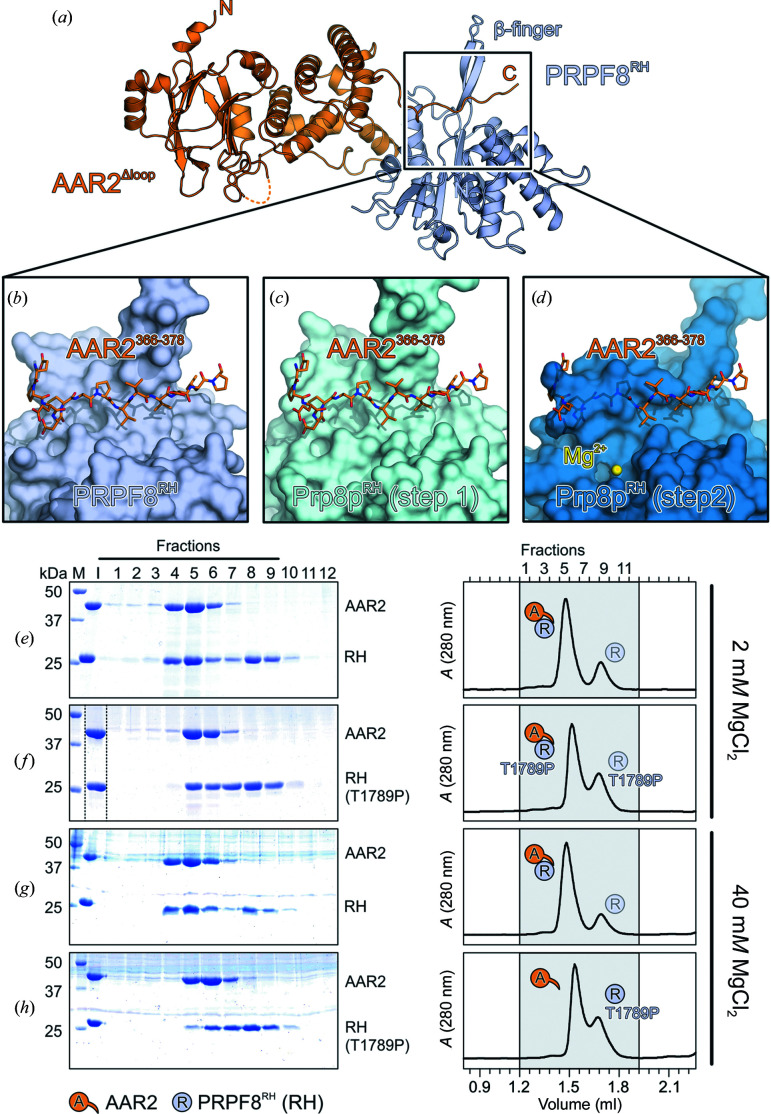
AAR2-mediated blocking of a step 2 conformation in PRPF8^RH^. (*a*–*d*) Structure of the AAR2^Δloop^–PRPF8^RH^ complex (*a*) and close-up views comparing the AAR2^Δloop^ C-terminal tail (sticks) traversing the PRPF8 RH domain below the protruding β-finger module (surface views) as observed in the AAR2^Δloop^–PRPF8^RH^ complex (*b*) or modeled onto the PRPF8^RH^ domain in the step 1 conformation (PDB entry 4jk7; Schellenberg *et al.*, 2013 ▸) (*c*) or onto the PRPF8^RH^ domain in the step 2 conformation (PDB entry 4jk7; Schellenberg *et al.*, 2013 ▸) (*d*) by superposition of the RH domains. Yellow sphere, coordinated Mg^2+^ ion. The AAR2 C-terminus clashes with the PRPF8^RH^ domain in the step 2 conformation. (*e*–*h*) SDS–PAGE analyses (left) and UV elution profiles (right) of analytical size-exclusion chromatography runs monitoring the interaction between AAR2 and PRPF8^RH^ (*e*, *g*) or PRPF8^RH,T1789P^ (*f*, *h*) at 2 m*M* (*e,* *f*) or 40 m*M* (*g*, *h*) magnesium chloride. Lane M, molecular-mass standard (kDa); lane I, input samples. Protein bands are identified on the right. Elution fractions are indicated at the top of the gel and profile in (*e*); elution volumes are indicated at the bottom of the profile in (*h*). Icons are explained at the bottom left. Variants are indicated below the respective icons. Peaks labeled with transparent icons represent an excess of the respective protein.

Comparison of our AAR2^Δloop^–PRPF8^RH^ structure with the PRPF8^RH^ step 1 and step 2 conformations revealed that AAR2 binding is compatible with the PRPF8^RH^ step 1 conformation but that steric clashes ensue between the AAR2 C-terminal tail and the PRPF8 RH domain in the Mg^2+^-bound step 2 conformation (Figs. 4 ▸ *a*–4 ▸ *d*). To test whether AAR2 is likely to prevent a switch of PRPF8^RH^ into the step 2 conformation, we explored whether AAR2 binds a PRPF8^RH^ variant that is stabilized in the step 2 conformation (T1789P; Schellenberg *et al.*, 2013 ▸). Indeed, unlike WT PRPF8^RH^, PRPF8^RH,T1789P^ partly dissociated from AAR2 upon increasing the Mg^2+^ concentration in analytical SEC (Figs. 4 ▸ *e*–4 ▸ *h*), suggesting that a step 2 conformation in PRPF8^RH^ is incompatible with AAR2 binding.

## 4. Discussion

We have elucidated similarities and differences in the structures and interaction profiles of yeast Aar2p and human AAR2 and have identified a putative, conserved phosphorylation event that is most likely to be involved in the functional cycle of AAR2 as a U5 snRNP assembly factor. Based on our findings, we conclude that the precise roles of Aar2p and AAR2 in U5 snRNP biogenesis differ. In yeast, an Aar2p–pre-U5 snRNP, from which the Brr2p RNA helicase is excluded, seems to constitute an important U5 snRNP assembly intermediate (Boon *et al.*, 2007 ▸; Weber *et al.*, 2013 ▸). In contrast, our observations of (i) human AAR2 failing to sequester the PRPF8 JM domain from SNRNP200^395–2136^ and (ii) AAR2 concomitantly binding to a PRPF8 fragment encompassing the RH and JM domains and SNRNP200^395–2136^ suggest that an equivalent, long-lived intermediate is not formed in the human system. Association of AAR2 with the PRPF8 RH domain as in our AAR2^Δloop^–PRPF8^RH^ structure would prevent the PRPF8 RH domain from engaging with other regions of PRPF8, the N-terminal region of SNRNP200, the C-terminal region of PRPF31, PRPF6, U4/U6 di-snRNAs and U5 snRNA as observed in the human U4/U6-U5 tri-snRNP (Agafonov *et al.*, 2016 ▸; Charenton *et al.*, 2019 ▸). This finding suggests that prevention of the premature association of U4/U6 di-snRNP components with pre-U5 particles may be an important function of AAR2 in the human system. In addition, transient blocking of binding sites on the PRPF8 RH domain, possibly supported by allosteric effects due to the selective stabilization of a step 1-like conformation in the PRPF8 RH domain by AAR2, may help to order assembly steps during U5 snRNP biogenesis. The above findings and suggestions are in agreement with the previous observation of the interaction of human AAR2 with a PRPF8–EFTUD2–SNRNP200–SNRNP40 U5 submodule (Malinová *et al.*, 2017 ▸).

As most protein-coding genes in humans contain multiple introns (Lee & Rio, 2015 ▸), pre-mRNA splicing is an inherent step in their expression. Moreover, pre-mRNA splicing predominantly occurs co-transcriptionally (Alpert *et al.*, 2017 ▸) and splicing is physically and functionally coupled to transcription, other pre-mRNA processing steps and mRNA export (Carrocci & Neugebauer, 2019 ▸; Tellier *et al.*, 2020 ▸). Thus, efficient splicing is a prerequisite for efficient gene expression and, due to its stabiliziation of the step 1 configuration of RH, a potential role of human AAR2 in pre-mRNA splicing cannot be ruled out. AAR2 may have a moonlighting function during pre-mRNA splicing independent of its role as a U5 snRNP assembly factor. By binding the PRPF8 RH domain during a stage of splicing when it is available, for example, in the pre-catalytic B complex (PDB entry 7abg; Townsend *et al.*, 2020 ▸), AAR2 may hinder the transition to subsequent stages, thus impeding splicing and, as a consequence, gene expression. As in the case of U5 snRNP assembly, direct blocking of binding sites on PRPF8^RH^ and allosteric effects due to the stabilization of a step 1 conformation in PRPF8^RH^ may support such a splicing-inhibitory role of AAR2. Again, the observed high nuclear levels of AAR2 might ensure that sufficient AAR2 is available to serve multiple functions, as moonlighting is known for some splicing factors that are in excess over other splicing machinery. For example, U1 snRNP has additional roles in 3′-end processing of Pol II transcripts (telescripting; Di *et al.*, 2019 ▸). However, AAR2 has never been found to be associated with the spliceosome at any stage of splicing (Agafonov *et al.*, 2011 ▸), arguing against a direct effect of AAR2 on splicing. Further studies on human Aar2 in a spliceosomal context will hopefully resolve these remaining questions.

## 5. Data availability

Structure coordinates and diffraction data have been deposited in the Protein Data Bank (http://www.pdb.org) under accession code 7pjh. All other data supporting the findings of this study are described in the manuscript or in the supporting information or are available from the corresponding authors on request.

## 6. Related literature

The following references are cited in the supporting information for this article: Barton (1993 ▸), Kabsch & Sander (1983 ▸) and Pettersen *et al.* (2004 ▸).

## Supplementary Material

PDB reference: AAR2 bound to PRPF8 RNaseH domain, 7pjh

Supplementary Figures. DOI: 10.1107/S2059798322009755/cb5141sup1.pdf

## Figures and Tables

**Table 1:** Crystallographic data (Santos *et al.*, 2015 ▸) and refinement Values in parentheses are for the highest resolution shell.

Data collection	
Wavelength (Å)	1.24
Temperature (K)	100
Space group	*C*2
*a*, *b*, *c* (Å)	145.28, 57.26, 111.23
α, β, γ (°)	90, 112.74, 90
Resolution (Å)	99.90–2.35 (2.41–2.35)
Unique reflections	34687 (2502)
Completeness (%)	97.6 (95.6)
Multiplicity	3.8 (3.9)
〈*I*/σ(*I*)〉	15.6 (1.7)
*R*_merge_(*I*)†	0.05 (0.92)
CC_1/2_ ‡	99.9 (76.5)
Refinement	
Resolution (Å)	48.21–2.35 (2.42–2.35)
Total No. of reflections	34678 (2667)
Completeness (%)	97.7 (95.7)
Reflections in test set (%)	4.96 (4.87)
*R*_work_ §	0.189 (0.347)
*R*_free_ ¶	0.235 (0.418)
ESU†† (Å)	0.35
Contents of asymmetric unit	
Protein molecules/residues	2/574
Water oxygens	147
Mean *B* factors (Å^2^)	
Wilson	55.39
Protein	77.81
Solvent	65.02
Ramachandran plot‡‡ (%)	
Favored	95.58
Allowed	4.42
Outliers	0
R.m.s.d.§§ from target geometry	
Bond lengths (Å)	0.009
Bond angles (°)	0.990
Dihedral angles (°)	115.920
PDB code	7pjh

† *R*_merge_(*I*) = 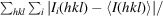 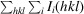 for *i* observations of a given reflection *hkl*; 〈*I*(*hkl*)〉 is the average intensity of the *i* observations.

‡ CC_1/2_ = (〈*I* ^2^〉 − 〈*I*〉^2^)/(〈*I* ^2^〉 − 〈*I*〉^2^) + 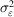 , where 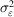 is the mean error within a half data set (Karplus & Diederichs, 2012 ▸).

§ *R*_work_ = 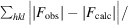 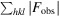 (for the working set; no σ cutoff was applied).

¶ *R*_free_ is the same as *R*_work_ but calculated on the test set of reflections that were excluded from refinement.

†† Estimated overall coordinate error based on maximum likelihood.

‡‡ Calculated with *phenix.refine* (Afonine *et al.*, 2012 ▸).

§§ Root-mean-square deviation.
