# Supplementary material for: Structural and functional investigation of the human snRNP assembly factor AAR2 in complex with the RNase H-like domain of PRPF8
Source: Acta Crystallogr D Struct Biol. 2022 Oct 27;78(Pt 11):1373–83. doi: 10.1107/S2059798322009755 (PMC9629490; doi:10.1107/S2059798322009755)
Supplement: Supplementary file 1 [file d-78-01373-sup1.pdf]

Acta Cryst D

STRUCTURAL BIOLOGY

Volume 78 (2022)

Supporting information for article:

Structural and functional investigation of the human snRNP assembly factor AAR2 in complex with the RNase H-like domain of PRPF8

Marco Preussner, Karine F. Santos, Jonathan Alles, Christina Heroven, Florian Heyd, Markus C. Wahl and Gert Weber

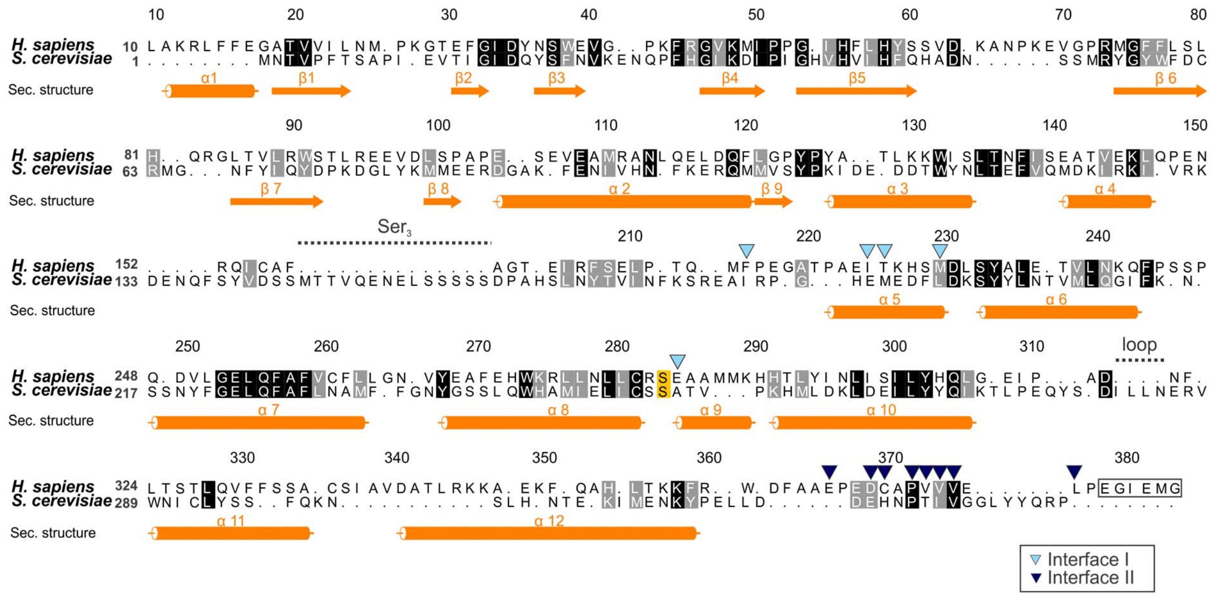

Figure S1 Structure-based alignment of yeast Aarp2 and structured parts of human AAR2.The alignment is based on separate superposition of the Aar2p and AAR2 NTDs, CTDs and C-terminal tails. The alignment was prepared with Chimera [1] and shaded with ALSCRIPT [2]. Identical and similar residues are indicated by black and gray backgrounds, respectively. The numbering above the alignment refers to human AAR2. Secondary structure elements of AAR2$^{\Delta loop}$ in the AAR2$^{\Delta loop}$-PRPF8^{RH} complex structure analyzed with DSSP [3] are shown below the alignment. The first ten unstructured residues of AAR2 are not shown in the alignment. Dashed lines indicate residues of AAR2 (not included in the alignment) that were replaced by three serine residues (Ser_3) in AAR2$^{\Delta loop}$ or residues 286-304 that lacked electron density, also not shown in the alignment (loop). Light blue (Interface I) and dark blue (Interface II) triangles above the alignment indicate residues of AAR2$^{\Delta loop}$ in contact with PRPF8^{RH}. The last six residues of AAR2$^{\Delta loop}$ (boxed) also lacked electron density and were not modeled.

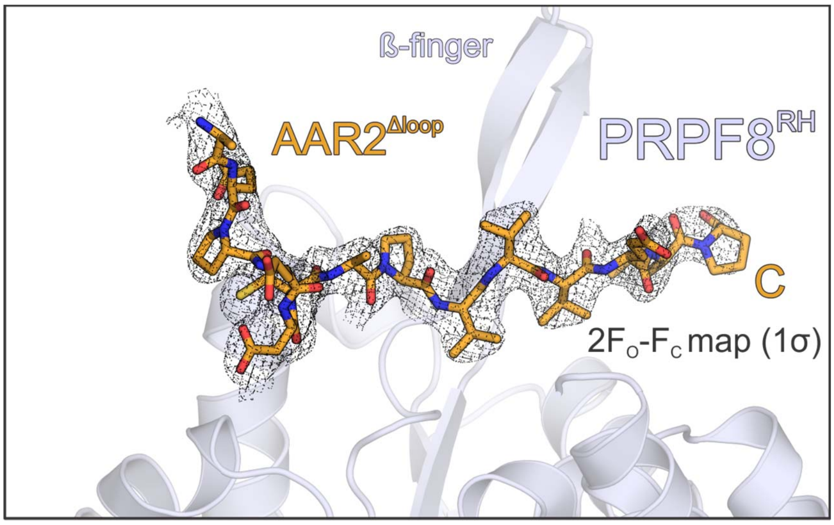

Figure S2 Electron density of the AAR2 C-terminal peptide. $2F_O$-F_C electron density around the human AAR2$^{\Delta loop}$ C-terminal tail (sticks) contoured at the 1 σ level.

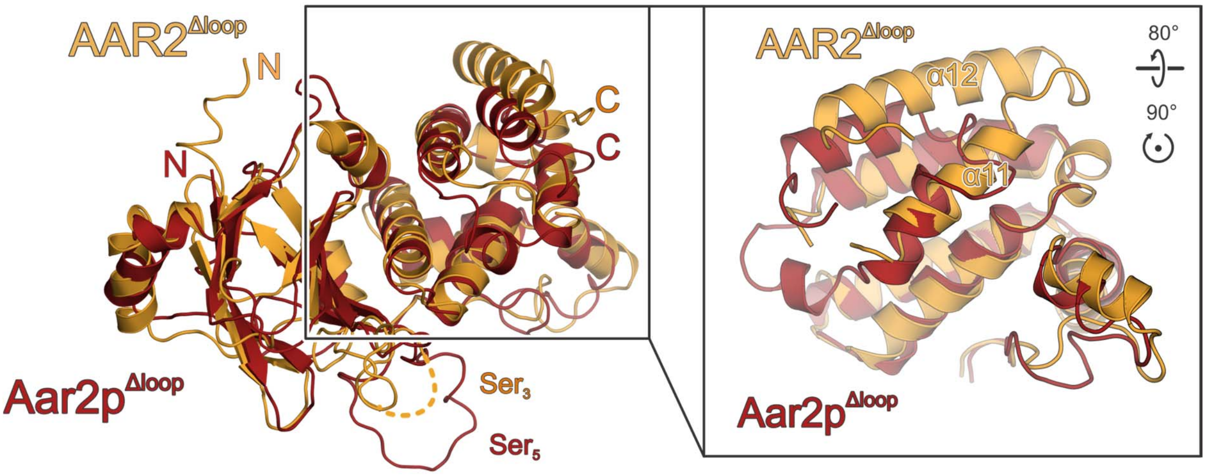

Figure S3 Supplementary Figure S3. Comparison of yeast Aar2p$^{\Delta loop}$ and human AAR2$^{\Delta loop}$. Superposition of yeast Aar2p$^{\Delta loop}$ (PDB ID 4ILG) [4] and human AAR2$^{\Delta loop}$, aligned by their CTDs. Inset, close-up view of the C-terminal domains showing the extended helices α11 and α12 of AAR2.

SUPPLEMENTARY REFERENCES

1. Pettersen, E.F., Goddard, T.D., Huang, C.C., Couch, G.S., Greenblatt, D.M., Meng, E.C., and Ferrin, T.E. (2004). UCSF Chimera - A visualization system for exploratory research and analysis. J. Comput. Chem. *25*, 1605–1612.
2. Barton, G.J. (1993). Alscript: A tool to format multiple sequence alignments. Protein Eng. Des. Sel. *6*, 37–40.
3. Kabsch, W., and Sander, C. (1983). Dictionary of protein secondary structure: Pattern recognition of hydrogen-bonded and geometrical features. Biopolymers *22*, 2577–2637.
4. Weber, G., Cristão, V.F., Santos, K.F., Jovin, S.M., Heroven, A.C., Holton, N., Lührmann, R., Beggs, J.D., and Wahl, M.C. (2013). Structural basis for dual roles of Aar2p in U5 snRNP assembly. Genes Dev. *27*, 525–540.
